# Postoperative Bleeding After Dental Implant Surgery in Patients on Direct Oral Anticoagulants: A Retrospective Case–Control Study

**DOI:** 10.3390/dj13120576

**Published:** 2025-12-03

**Authors:** Yifat Manor, Gil Ben-Izhack, Paul Alexander Manor, Oren Peleg, Shoshana Reiter

**Affiliations:** 1 Dental Department Shamir Medical Center, Beer Yaacov 7033001, Israel; gil.ben@shamir.gov.il; 2 Department of Oral and Maxillofacial Surgery, School of Dental Medicine, Gray Faculty of Medical and Health Sciences, Tel Aviv University, Tel Aviv 69978, Israel; orenpeleg@tauex.tau.ac.il; 3 Economical Unit Edith Wolfson Medical Center, Holon 58100, Israel; amanor@wmc.gov.il; 4 Department of Oral and Maxillofacial Surgery, Sourasky Medical Center, Tel Aviv 642390, Israel; 5 Department of Oral Radiology, Oral Medicine, Oral Pathology, School of Dental Medicine, Gray Faculty of Medical and Health Sciences, Tel Aviv University, Tel Aviv 69978, Israel; shoshanareiter@tauex.tau.ac.il

**Keywords:** dental implantation, complications, bleeding, direct oral anticoagulants (DOACs), bone grafting, drug holiday

## Abstract

**Background**: Dental implants are popular among both healthy and medically complex patients. Patients prone to thromboembolic events receive anticoagulant treatment, like direct oral anticoagulants (DOACs). When those patients undergo dental implantation, the risk of bleeding is higher. **Objectives**: This study primarily aims to describe and analyze bleeding events following dental implantation. The secondary aim is to identify risk factors for bleeding events in patients receiving DOACs, and to suggest precautions. **Methods**: A case–control retrospective study was carried out on patients who underwent dental implant insertion in a single dental clinic. The experimental group comprised patients on DOACs, while the control group consisted of healthy patients without anticoagulant treatment. The study was submitted and approved by the Helsinki committee of the medical center (ASF-205-23). **Results**: Out of 100 patients initially screened for the study, 80 were included: 41 in the experimental group and 39 in the control group. A total of 11 patients presented with bleeding: 7 in the experimental group and 4 in the control group. A higher incidence of bleeding was observed in the experimental group (17%) compared with the control group (10%). The odds of bleeding in patients receiving DOACs were 1.8 times higher than for those not receiving anticoagulant therapy. Bleeding risk was also elevated among patients who underwent bone grafting procedures, with an odds ratio (OR) of 3.47, and among patients aged over 75 years, with an OR of 3.92. All bleeding events were successfully managed using local hemostasis. **Conclusions**: Despite the study’s limitations, it can be concluded that surgeons should consider complex surgical intervention, but should be careful and aware of several risk factors and precautions regarding bleeding. Patients on DOACs with additional bone grafting and medically compromised patients over 75 years old should be monitored in the first postoperative days, and the postoperative guidelines should be refined to address the risks effectively.

## 1. Introduction

Dental implants have become very popular among both healthy and medically compromised patients. Patients on anticoagulant treatment for various conditions may also need dental implants to restore missing teeth. Previously, patients requiring parenteral anticoagulant received heparin, while those needing oral anticoagulation were treated with warfarin [1]. However, due to warfarin’s narrow therapeutic index and the need for frequent laboratory monitoring, direct oral anticoagulants (DOACs) were subsequently developed [2].

DOACs include direct thrombin inhibitors (e.g., dabigatran) and factor Xa inhibitors (e.g., rivaroxaban, apixaban, edoxaban), which are designed to target different points of the coagulation cascade.

### Direct Oral Anticoagulants (DOACs)

Direct factor Xa inhibitors like apixaban (eliquis), rivaroxaban (xarelto), and edoxaban (lixiana) and direct thrombin inhibitors like dabigatran (pradaxa) are alternatives for preventing blood clot formation in selected patients. Unlike warfarin, DOACs do not require regular laboratory monitoring and are not affected by food or alcohol. DOACs have more predictable pharmacokinetic profiles than warfarin, along with shorter half-lives and less need for routine laboratory monitoring.

Practical guidelines [3] for performing oral surgical procedures in patients taking DOACs recommend that, for intervention with low bleeding risk where temporary reduction in anticoagulation is desired, DOAC therapy should be withheld the day before surgery and resumed the day after the procedure. For procedures with a high bleeding risk, DOACs are withheld for 2 days prior to surgery and can be resumed 2 to 3 days after surgery.

The primary aim of this study is to describe and analyze bleeding events in patients following dental implantation, based on clinical data collected from a single dental clinic within a medical center. The secondary aim is to identify risk factors for bleeding and to suggest precautions for dental implant insertion in patients on DOACs.

The literature discusses complications in patients receiving DOACs and other anticoagulants. Dawoud et al. [4] conducted a meta-analysis investigating the effect of continuing with anticoagulant therapy during dental implant surgery and found an increased risk of bleeding. They recommended that the decision to discontinue anticoagulants should be based on a balanced assessment of bleeding and thromboembolic risks. Others (Gomez-Moreno [5,6]) did not find any difference in bleeding between patients receiving dabigatran or rivaroxaban without modification of the treatment. It was found [7] that those patients can undergo dental implantation safely when treated with other antithrombotic drugs, like antiplatelet drugs. Regarding the need to cease medical treatment using medications others than DOACs, it was found [8] that in the cases of vitamin K and non-vitamin K antagonists, antiplatelet agents, and low-molecular-weight heparin K antagonist, the absolute risk is low and there is no need to discontinue or alter the antithrombotic treatment dose for implant placement. The risk of bleeding regarding all kinds of antithrombotic agents [9] was low, and no alteration in medical treatment was recommended.

Bleeding events in patients receiving DOAC therapy have been analyzed across various types of dental procedures [10]. It was concluded that most dental treatments can be performed without a significant increase in bleeding risk, regardless of the anticoagulant discontinuation duration. Moreover, any postoperative bleeding was effectively managed using compressive pressure or local hemostatic agents.

Clemm et al. [11] evaluated the postoperative bleeding risk of patients continuing their anticoagulation therapy and undergoing implant surgery and bone grafting procedures and found 1.2% of postoperative bleeding in a group taking antiplatelet drugs, vitamin K antagonists, and DOACs.

A meta-analysis [12] reviewed seven articles on the use of anticoagulants or anti-platelet aggregation and found a higher tendency for bleeding in patients receiving vitamin K antagonists but not in patients receiving DOACs.

This study analyzed cases of bleeding events following dental implantation and bone grafting in patients on DOACs (rivaroxaban (xarelto), apixaban (eliquis), and dabigatran (pradaxa) based on a single-center study. The null hypothesis is that patients on DOACs do not experience a higher incidence of bleeding events compared to the general population, and that bleeding occurrences can be minimized through the implementation of a simple treatment protocol.

## 2. Patients and Methods—Study Design

This was a case–control retrospective study on patients who were treated at a single dental clinic within a medical center by trained specialists of oral and maxillofacial surgery between the years 2019 and 2022. The study was conducted in accordance with the STROBE (Strengthening the Reporting of Observational Studies in Epidemiology) guidelines (Table S1).

The study was approved by the Helsinki committee of the institution (ASF-205-23; approval date on 27 November 2023). Due to institutional and national regulation of retrospective studies, patient informed consent to participate in the study was waived by the board.

### 2.1. Inclusion Criteria

Patients requiring dental implant insertion in one quadrant of the mouth.

Experimental group: Patients on DOACs for at least 6 months prior to implant surgery.

Control group: Patients not taking any anticoagulants or antiplatelet medications.

Patients who had undergone dental implant insertion by a specialist of oral and maxillofacial surgery with at least 10 years’ experience.

Patients were included if their medical records contained complete demographic and clinical data, documentation of any bleeding events verified visually and managed during the 14-day postoperative period, and confirmation of a completed clinical follow-up visit at 14 days after surgery.

### 2.2. Exclusion Criteria

Patients with bleeding disorders.

Patients with uncontrolled medical status.

Patients with a history of radiation therapy to the head and neck region.

Patients without follow-up.

Patients with missing data in their medical files.

**Figure 1:**
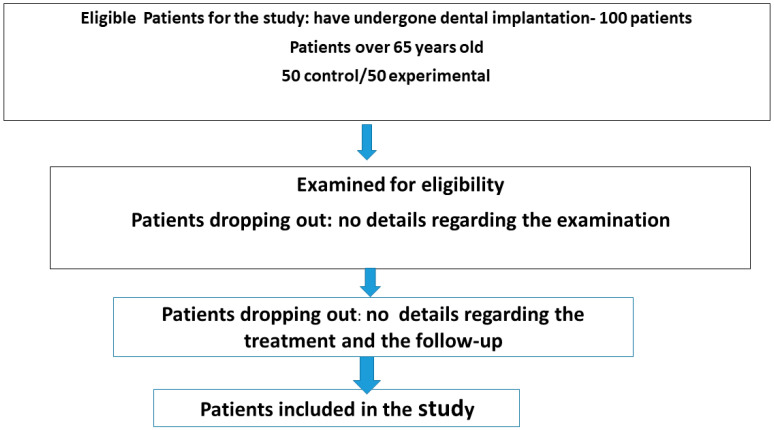
A flow diagram of patients in the study.

Figure 1 presents a flow diagram of the patients included in the study.

To minimize age-related bias, the lower age limit for inclusion in both the experimental and control groups was set at 65 years. The control group included systemically healthy patients (ASA I) who underwent dental implant placement and were age-matched to the DOAC group. No matching was performed for systemic condition or ASA classification, as DOAC therapy is prescribed to patients with systemic comorbidities corresponding to ASA II–III. All the analyzed cases included submerged implants; cases used allografts and xenografts where needed.

The experimental group consisted of patients treated with DOACs before the study (e.g., rivaroxaban, dabigatran, and apixaban).

### 2.3. Treatment Protocol

Before dental implant placement, all patients on DOAC therapy consulted their physician to assess whether a 24–48 h drug holiday was appropriate, balancing the perioperative bleeding risk against the risk of thromboembolism. All procedures were performed under local anesthesia, following preoperative rinsing with 0.2% chlorhexidine. Antibiotic prophylaxis was administered according to the American Heart Association (AHA) recommendations and the institutional protocol.

Local hemostasis was achieved using absorbable sutures. A maximum of three implants were inserted per session, confined to a single quadrant. In cases requiring bone grafting, a full-thickness flap was elevated with releasing incisions to allow implant placement and bone augmentation. Primary flap closure was achieved using interrupted monofilament sutures. In the posterior maxilla, when the residual bone height was limited (5–8 mm), a sinus floor augmentation procedure was performed. All implants were submerged throughout the whole healing process.

Bleeding was assessed objectively, both during and after the surgical procedure. Postoperative evaluation was performed 20–30 min after completion of surgery, and continued for at least 30 min, by two independent members of the clinical staff. At the end of surgery, patients were instructed to apply firm pressure on the surgical site by biting on sterile gauze for 20–30 min. Additionally, all patients were advised to apply external ice packs to the surgical area for 1–3 h postoperatively. Postoperative instructions included maintaining a cold, soft diet, avoiding mastication on the operated side, and refraining from wearing removable dentures for up to two weeks when there was a risk of pressure or impingement on the surgical site. Bleeding events were documented based on standardized visual assessment.

Patients were instructed to monitor for any postoperative bleeding and to return to the clinic if bleeding occurred for additional hemostatic management. Bleeding status was evaluated based on the presence or absence of bleeding at the time of examination. For each bleeding event, the required hemostatic interventions were recorded and summarized. Emergency management of bleeding included additional suturing in cases of mild bleeding or oozing from the incision line. In cases of moderate bleeding, additional suturing was combined with the application of compressive gauze soaked in tranexamic acid. All supplementary sutures were placed using silk sutures.

Antibiotic and analgesic treatment: Antibiotic prophylaxis was administered 1 h before surgery according to the institutional protocol (amoxicillin 1 g). Postoperatively, patients undergoing bone grafting who were not allergic to penicillin received amoxicillin 500 mg three times daily for 5–7 days; penicillin-allergic patients received azithromycin 500 mg once daily for 3 days. This protocol reflects local clinical practice to reduce the risk of operative infection in bone grafting procedures.

For patients meeting the American Heart Association indications for endocarditis prophylaxis, a single preoperative dose of amoxicillin 2 g was used, or azithromycin 500 mg for those with penicillin allergy, both administered 1 h before surgery. Analgesics prescribed included paracetamol (Acamol), dipyrone (Optalgin), and naproxen sodium (narocin), administrated as clinically indicated.

Sample size calculation: The data were collected from the records of all patients treated by experienced maxillofacial surgeons at a single medical center. All eligible cases within the study period were included.

Statistical analyses were performed using SPSS version 29.0 for Windows. The level of significance was set at *p* < 0.05. Descriptive statistics were used to summarize the data, and comparative analyses were conducted using the Chi-square test or Fisher’s exact test for categorical variables. Bleeding events were analyzed as a binary outcome (presence or absence of postoperative bleeding). Group comparisons were performed using the Chi-square test, or Fisher’s exact test when expected frequencies were <5. Absolute risks were calculated for each group, and results are presented as risk differences and odds ratios (ORs) with 95% confidence intervals (CIs).

## 3. Results

**Figure 2:**
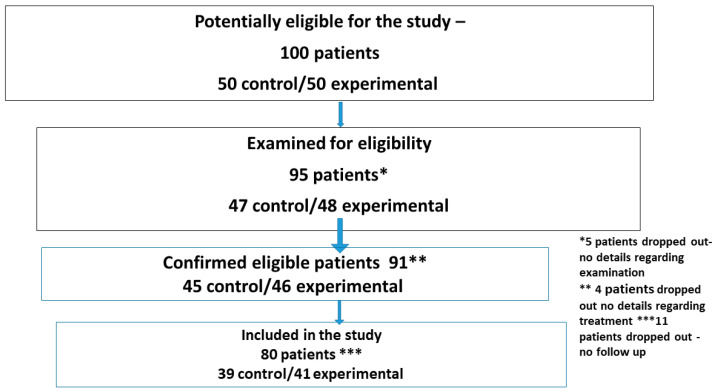
A flow chart of patients included in the study.

A total of 100 patients were initially considered for inclusion in the study. Of these, 95 were assessed for eligibility, 91 met the inclusion criteria, and 80 completed follow-ups and were included in the final analysis (Figure 2—flow diagram of patient selection).

Among the 80 patients included in the study, 41 were assigned to the experimental group and 39 to the control group. The mean ages were 73 years (range: 65–90 years) and 72 years (range: 66–87 years), respectively.

The medical status of the experimental group, assessed according to the American Society of Anesthesiologists (ASA) classification, included 19 patients classified as ASA II and 22 as ASA III. All patients in the control group were systemically healthy (ASA I).

**Figure 3:**
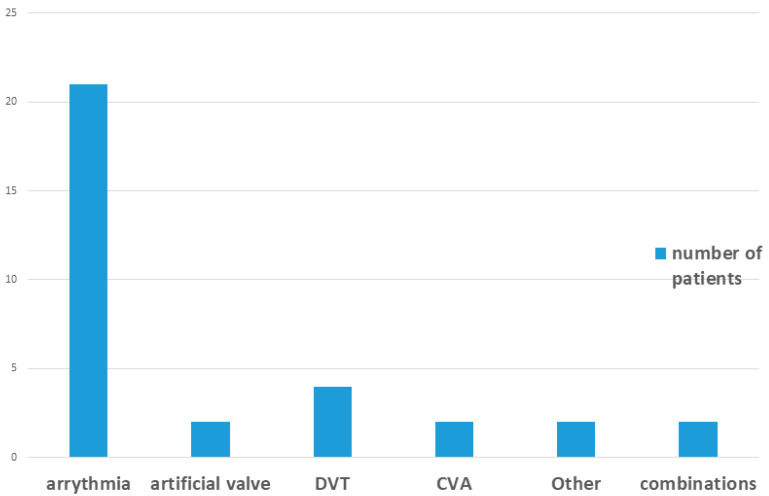
Clinical diagnosis for anticoagulant therapy.

Among patients receiving DOAC therapy, 38 were treated with apixaban (Eliquis^®^Teva, Petah Tikva, Israel) or rivaroxaban (Xarelto^®^ Janssen Pharmaceuticals, Titusville, NJ, USA), and 3 with dabigatran (Pradaxa^®^ Boehringer, Ingelheim, Germany). None of the control group patients were receiving anticoagulant therapy. Additional details are provided in Table 1 and Figure 3.

Treatment protocol: Among patients receiving DOACs, perioperative management was determined in coordination with each patient’s physician. Of the 41 patients, 7 (17.1%) continued their anticoagulant therapy during the surgical procedure, while the remaining 34 (82.9%) temporarily discontinued medication 24–48 h before surgery, in accordance with their physician’s recommendations. No bridging therapy was administered.

### 3.1. Postoperative Bleeding Outcomes

Bleeding events occurred in 11 patients across both groups. Seven cases were successfully managed with additional sutures and local pressure. In the control group, four patients experienced postoperative bleeding: two were older than 75 years, and two were younger than 75 years. Bleeding events were more frequent in females and in patients who underwent bone grafting; the age distribution (>75 vs. <75) was equal. In the DOAC group, there was a trend toward more bleeding in patients over 75 years and in those who had dental implantation with bone grafting. Implant site (anterior versus posterior), jaw (maxilla versus mandible), and type of DOAC (dabigatran, rivaroxaban, apixaban) had no significant effect on bleeding events. Overall, the proportion of patients who bled was higher in the anticoagulant group than in the control group, but this difference was not statistically significant. All bleeding episodes in both groups were managed with local measures; nonabsorbable silk sutures were placed to maintain wound-edge tension and support prolonged hemostasis during the postoperative period. Clinical details for bleeding and non-bleeding patients in both groups are presented in Table 2a,b and Table 3.

### 3.2. Statistical Analysis

To test the hypothesis that patients on DOACs tend to experience more bleeding than those who are not, a Chi-square test and Fisher’s exact test were used. Postoperative bleeding occurred in 7 of 41 patients in the DOAC group (17.1%) and in 4 of 39 controls (10.3%). The difference was not statistically significant (Fisher’s exact test, *p* = 0.52; OR = 1.80, 95% CI 0.48–6.72). We used the odds ratio to express the trend, and the odds ratio for bleeding in patients on DOACs was 1.8.

A connection was found between bleeding and concomitant bone grafting with dental implant insertion. The odds ratio for bleeding associated with dental implantation with bone grafting was 3.47, meaning these patients were approximately 3.5 times more likely to experience postoperative bleeding than those who underwent implantation without bone grafting.

The main risk factors for bleeding in both groups were age over 75 years (odds ratio 3.92) and medical compromise, especially ASA 3 status (odds ratio 2.57).

Subgroup analyses were performed to evaluate the impact of DOACs management strategy (continued vs. temporarily paused) and the presence of bone grafting on postoperative bleeding. Categorical comparisons were assessed using Fisher’s exact test (2 × 2 contingency), and where applicable, χ^2^ tests with reported degrees of freedom (df = 1). The corresponding odds ratios with 95% confidence intervals (CIs) were calculated. Among the 41 patients treated with DOACs, 7 (17.1%) continued therapy and 34 (82.9%) paused medication prior to surgery.

Bleeding occurred in 2 of 7 (28.6%) patients who continued DOACs and 5 of 34 (14.7%) who paused medication. The difference was not statistically significant (Fisher’s exact test, df = 1, *p* = 0.58; OR = 2.29, 95% CI 0.35–14.85).

Bleeding occurred in 5 of 23 patients (21.7%) who underwent bone grafting and in 2 of 18 patients (11.1%) who received implant placement alone. The absolute risk difference was 10.6% (95% CI—11.6% to 32.9%), and the risk ratio was 1.96 (95% CI 0.44–6.84). Although bleeding was more frequent in grafting cases, the difference did not reach statistical significance (Fisher’s exact test, df = 1, *p* ≈ 0.44).

Other parameters like site of operation, number of implants per procedure, gender and type of DOAC did not significantly affect postoperative bleeding.

### 3.3. Clinical Implications

The study indicates that patients taking DOACs may have an increased risk of postoperative bleeding after dental implantation—particularly when bone grafting is performed, when no perioperative drug interruption is observed, and in medically compromised patients aged over 75 years.

In cases of extensive surgical intervention, there are several steps that should be taken to minimize the bleeding events: Drug holiday—this should be carefully considered in consultation with the patient’s physician regarding the risk of thromboembolic events. Close monitoring during the first postoperative days is recommended, particularly after extensive surgical procedures. Enhanced surveillance should focus on patients with identified risk factors—those on DOACs undergoing implantation with bone grafting, medically compromised individuals, and patients older than 75 years. Refinement of postoperative guidelines is advised to address these risks more effectively, for example, by specifying follow-up timing, hemostatic measures, and individualized plans for perioperative anticoagulant management.

## 4. Discussion

Dental implantation with bone augmentation is commonly performed and is not routinely contraindicated in patients receiving oral anticoagulants. All surgical procedures carry some risk of intraoperative and postoperative bleeding, a risk that is increased in anticoagulated patients. Intraoral hemorrhage can be particularly hazardous because it may compromise both oral intake and airway patency, so prevention and rapid control are essential. This study reports our clinic’s experience managing patients on direct oral anticoagulants (DOACs) and identifies clinical factors associated with bleeding. DOACs have relatively short half-lives and lack widely available laboratory tests that reliably predict bleeding risk, which complicates perioperative decision making. Although we observed a higher proportion of bleeding in the DOAC group, the difference between groups was not statistically significant; the estimated odds ratio for bleeding in DOAC-treated patients was 1.8. By contrast, implantation combined with bone grafting showed a larger association with bleeding, and older age and higher ASA status also emerged as important risk factors. Published data are mixed regarding bleeding risk with individual DOAC agents; some reports suggest higher postoperative bleeding with rivaroxaban compared with controls, while others indicate a less pronounced effect with dabigatran [13]. In our case series, all bleeding events were successfully managed with local hemostasis, consistent with prior clinical experience that most oral bleeding on antithrombotic therapy can be controlled without systemic intervention [5,6,7,8,9].

Regarding the current consensus for DOACs, short molecule-specific interruption is often recommended for high-bleeding-risk procedures; for VKAs, decisions are guided by INR and thrombotic risk; for antiplatelet agents, continuation is often advised for single-agent therapy [14]. Several studies have examined the effect of a drug holiday from DOACs on postoperative bleeding. It was found [10] that most dental treatments can be performed in patients taking DOACs with no clear increase in bleeding risk regardless of the anticoagulant interruption duration. On the other hand, a drug holiday of 24 h was not associated with a significant change in postoperative bleeding following dental implantation with immediate loading [15]. Conversely, Hanken et al. [16] found that continuation of anticoagulation with the same agent significantly increased the risk of postoperative bleeding after oral surgical procedures; however, these bleeding events were manageable. Close monitoring for up to one week postoperatively was recommended to identify and treat any excessive bleeding.

A review of bleeding disorders and dental implants concluded that, with careful preoperative assessment and vigilant postoperative hemostatic management, patients receiving anticoagulant therapy can safely undergo dental implant placement [17].

The present study was not intended to check the correlation between postoperative bleeding and the continuation of anticoagulant treatment. For patients undergoing the procedure on a drug holiday, no thromboembolic events were reported to the authors. The decision to discontinue anticoagulants prior to dental implant surgery must consider both patient and surgical factors, with the clinician performing a risk-balance assessment. Bleeding occurred in 2 of 7 (28.6%) patients who continued DOACs and 5 of 34 (14.7%) who paused medication. The difference was not statistically significant. Nevertheless, the risk of bleeding should be explained to patients, and they should be instructed on methods to prevent and manage it; cases of moderate bleeding should be treated at the clinic using additional non-absorbable sutures and local pressure of tranexamic acid gauze. The surgeon should give special attention to cases involving bone grafting and provide patients with clear instructions for postoperative care.

### 4.1. Bone Grafting

This study found that the risk of bleeding was 3.47 times higher in patients undergoing bone grafting than in those receiving implant placement alone. A case–control study [18] of maxillary sinus elevation in patients on DOAC therapy also reported a greater frequency of bleeding in the anticoagulated group. The authors assume that the additional periosteal release often required for primary closure after grafting may account for the increased bleeding, compared with straightforward implant placement, which typically requires less extensive periosteal manipulation.

#### Types of DOACs

This study found no significant differences in postoperative bleeding among the various DOAC agents. Comparable studies [2] evaluating DOACs, antiplatelet therapies, and vitamin K antagonists have likewise reported no differences between specific DOACs; however, they identified a significantly higher rate of postoperative bleeding in the combined DOAC/antiplatelet group compared with the VKA group, a comparison not addressed in our study. Neither this study nor those published elsewhere observed any cases of severe bleeding. Our findings relate specifically to patients receiving DOACs and should not be generalized to all antithrombotic regimens. VKAs (e.g., warfarin) are monitored with INR and have established peri-operative management protocols that often balance thrombotic vs. bleeding risk. Different from DOACs, they require different reversal and bridging considerations. Antiplatelet agents (e.g., aspirin, clopidogrel) primarily affect platelet function and are managed according to different risk frameworks. DOACs differ from Vitamin K Antagonists in pharmacokinetics (shorter half-lives, predictable effect, limited routine monitoring) and in accessible reversal agents for some molecules; peri-operative recommendations for DOACs may therefore favor short, molecule-specific temporary interruption for higher-risk procedures rather than routine continuation or prolonged interruption.

### 4.2. Bleeding Events and Their Management

In this study, bleeding events occurred between postoperative days 1 and 3 in both groups. By contrast, a study of bleeding during dental hygiene procedures [19] reported shorter episodes of bleeding (98–120 s) in patients on DOACs controllable with local pressure.

We characterized postoperative bleeding in our cohort and found that mild hemorrhage was effectively managed with additional non-absorbable silk sutures and pressure dressings soaked with tranexamic acid. Possible contributors to the delayed bleeding timing include the use of absorbable sutures (which lose tensile strength as they resorb), resumption of DOACs approximately 12 h after surgery, and patients returning to normal oral function during the early postoperative days. Silk sutures were preferred because they maintain wound-edge tension longer than absorbable sutures, supporting prolonged hemostasis; no further bleeding was observed after applying these local measures. Use of topical tranexamic acid in the postoperative period is supported by the literature [20] and has been associated with a reduced bleeding risk.

More bleeding events were observed in medically compromised patients (OR = 2.57) and in patients older than 75 years (OR = 3.92); these differences did not reach statistical significance and warrant further investigation.

### 4.3. Study Limitations

This study has certain limitations. The DOAC group consisted of patients classified as ASA II–III, while the control group included only ASA I individuals. As DOAC therapy is generally prescribed for patients with systemic comorbidities, complete matching for ASA status was not feasible. Although groups were age-matched, the difference in overall health status may have introduced residual confounding that could have influenced bleeding outcomes.

The small number of bleeding events limited our ability to analyze causes of bleeding beyond DOAC exposure and reduced statistical power for subgroup analyses.

The modest sample size—only 80 eligible patients—may be insufficient to draw definitive conclusions or detect less common risk factors.

Our single-center design may restrict the generalizability of findings to other clinical settings and patient populations.

Retrospective data collection relied on existing records; cases with missing information for key variables were excluded, which could introduce selection bias.

Operator consistency—all procedures were performed by oral and maxillofacial surgeons with at least 10 years’ experience, which supports procedural quality but may limit applicability to less experienced operators.

Institutional antibiotic policy—routine postoperative antibiotics for 5–7 days reflects our local protocol and may not align with broader antibiotic stewardship guidelines.

## 5. Conclusions

Within the limitations of this study, the findings suggest that special attention should be given to medically compromised patients over 75 years of age who are receiving DOAC therapy and undergoing dental implant placement. Although a higher rate of postoperative bleeding was observed, the difference was not statistically significant, consistent with the known effects of anticoagulant therapy on bleeding risk. All bleeding events were successfully managed with local hemostatic measures in the dental clinic. However, more complex surgical procedures may require additional precautions and individualized perioperative planning.

Clinical Practice: Although this study provides valuable insights, the recommendations—such as the use of non-absorbable sutures and close postoperative monitoring of patients on DOAC therapy—should be validated through larger-scale studies and broader clinical experience.

## Figures and Tables

**Table 1:** Clinical and demographic details of the experimental and control groups.

	Experimental Group	Control Group
**Average age**	73 years old	72 years old
**Gender (M/F)**	27/14	20/19
**Upper jaw (no. of patients)**	15	20
**Lower jaw (no. of patients)**	26	19
**Dental implants (no. of patients)**	18	24
**Dental implants and bone grafting (no. of patients)**	23	15
**Bleeding cases**	7	4
**Patient age** **<75/>75**	20/21	35/4

**Table 2:** (**a**) Clinical and demographic details regarding the bleeding patients in the experimental and control groups. (**b**) Clinical and demographic details regarding the non-bleeding patients in the experimental and control groups.

	Experimental Group	Control Group
(**a**)		
**No. of bleeding patients**	7	4
**Gender (M/F)**	4/3	1/3
**Upper/lower jaw**	3/4	2/2
**Anticoagulant treatment** **rivaroxaban/apixaban**	1/6	0
**Drug holiday yes/no**	5/2	Not relevant
**Implants/implant and bone grafting**	2/5	1/3
**Patient age** **<75/>75**	2/5	2/2
(**b**)		
**No. of patients without bleeding**	34	35
**Gender (M/F)**	23/11	19/16
**Upper/lower jaw**	12/22	18/17
**Anticoagulant treatment** **rivaroxaban/apixaban/dabigatran**	12/26/3	0
**Implants/implant and bone grafting**	16/18	23/12
**Patient age** **<75/>75**	18/16	28/7

**Table 3:** Characteristics of bleeding patients: experimental and control groups.

Case Number	Age	Gender	Treated Area	Medical Treatment	Operation	Drug Holiday	Group	Method of Hemostasis
1	76	f	Upper jaw	rivaroxaban xarelto©	Implants and bone grafting	no	Experimental group	Additional suturing and local pressure tranexamic acid gauze
2	65	f	Lower jaw	apixaban eliquis©	Implants and bone grafting	yes	Experimental group	Additional suturing
3	65	m	Lower jaw	apixaban eliquis©	Implants and bone grafting	yes	Experimental group	Additional suturing
4	82	f	Lower jaw	apixaban© eliquis©	Implants and bone grafting	yes	Experimental group	Additional suturing and local pressure tranexamic acid gauze
5	80	m	Lower jaw	apixaban eliquis©	Implants	yes	Experimental group	Additional suturing and local pressure tranexamic acid gauze
6	76	m	Upper jaw	apixaban eliquis©	Implants	yes	Experimental group	Additional suturing and local pressure tranexamic acid gauze
7	86	m	Lower jaw	apixaban eliquis©	Implants and bone grafting	no	Experimental group	Additional suturing and local pressure tranexamic acid gauze
8	72	f	Lower jaw	none	Implants and bone grafting	no	Control group	Additional suturing
9	78	m	Lower jaw	none	Implants and bone grafting	no	Control group	Suturing and local pressure tranexamic acid gauze
10	71	f	Lower jaw	none	Implants and bone grafting	no	Control group	Suturing and local pressure tranexamic acid gauze
11	82	f	Lower jaw	none	implants	no	Control group	Additional suturing

## Data Availability

The datasets used and/or analyzed during the current study are available from the corresponding author on reasonable request.

## Supplementary Materials

The following supporting information can be downloaded at: https://www.mdpi.com/article/10.3390/dj13120576/s1, Table S1: STROBE Statement—Dental Implantation in Patients on Direct Oral Anticoagulants—A retrospective case control study.
