# Supplementary material for: Postoperative Bleeding After Dental Implant Surgery in Patients on Direct Oral Anticoagulants: A Retrospective Case–Control Study
Source: Dent J (Basel). 2025 Dec 3;13(12):576. doi: 10.3390/dj13120576 (PMC12732248; doi:10.3390/dj13120576)
Supplement: Supplementary file 1 [file dentistry-13-00576-s001.zip › dentistry-3893429-supplementary.pdf]

Table S1. STROBE Statement— **Dental Implantation in Patients on Direct Oral Anticoagulants – A retrospective case control study**

	item	recommendation	pp	how
**Title and abstract**	1	**(*a*) Indicate the study's design with a commonly used term in the title or the abstract**	1-2	Retrospective case control study
**Title and abstract**		(*b*) Provide in the abstract an informative and balanced summary of what was done and what was found	2-3	The abstract summarizes the manuscript
introduction	Background/rationale	Explain the scientific background and rationale for the investigation being reported	4	Enrichment of knowledge for dentists dealing with dental implantation
introduction	Objectives	State specific objectives, including any prespecified hypotheses	5	protocol of dental implant insertion in patients under DOAC
Study design		Present key elements of study design early in the paper	6-7	Retrospective case control study
Setting		Describe the setting, locations, and relevant dates, including periods of recruitment, exposure, follow-up, and data collection	6-7	Patients undergone dental implant insertion at a dental clinic in a single medical center
Participants		*Cohort study*—Give the eligibility criteria, and the sources and methods of selection of participants. Describe methods of follow-up	6-7	Retrospective case control study, age over 65 dental implantation by an experiences surgeon in healthy and under DOACs in a single center dental clinic, undergone treatment at

				one oral quadrant, documentation regarding bleeding events' bleeding events treated at the same dental clinic and follow up by visual check 7-14 days postoperatively, only full data cases were included in the study<br>Cases without full data were not included
		) *Cohort study*—For matched studies, give matching criteria and number of exposed and unexposed	6-8	Experimental group- patients undergone dental implant insertion under DOACs<br>Control group- patients with the same demographic and clinic parameters not under any anticoagulants
variables		Clearly define all outcomes, exposures, predictors, potential confounders, and effect modifiers. Give diagnostic criteria, if applicable	7-8	Bleeding yes/ no, area of dental implantation , way of hemostasis yes/no<br>Bone grafting yes/no<br>Medical status by ASA, age over/under 75 years old
Data sources/ measurement		For each variable of interest, give	7-8	Data from patients under

		sources of data and details of methods of assessment (measurement). Describe comparability of assessment methods if there is more than one group		follow up at a single center dental clinic , cases of bleeding were evaluated and treated in the clinic, assessment of surgical wound after 7-14 days, assessment of bleeding, assessment of bleeding events during the follow up peiod, assessment of hemostatic methods
Bias		Describe any efforts to address potential sources of bias	7-8	Patients were treated by an experienced specalist of oral and maxillofacial surgery under similar protocol of treatment evaluation of bleeding was assessed by two independent people of the staff
Study size		Explain how the study size was arrived at	9	The study **siz**e was determined according to 30 patients in each group and DF for each variable(age, gender, selection of a higher number and exclusion those who did not meet the full criteria to be included

Quantitative variables	11	Explain how quantitative variables were handled in the analyses. If applicable, describe which groupings were chosen and why	8-9	The only quantitative variable included in the analysis was **patient age**. For descriptive purposes, it was additionally categorized above in below 75 years old
Statistical methods		(*a*) Describe all statistical methods, including those used to control for confounding	9	descriptive Statistics, comparative Analysis: , Chi-square or Fisher's exact tests for categorical variables. Regression Analysis: logistic regression to identify independent risk factors for bleeding, adjusting for potential confounders
		(*b*) Describe any methods used to examine subgroups and interactions	9	chi square test for correlation between variables
		(*c*) Explain how missing data were addressed	8-9	Patients were not included in the study
		(*d*) *Cohort study*—If applicable, explain how loss to follow-up was addressed *Case-control study*—If applicable, explain how matching of cases and controls was addressed *Cross-sectional study*—If applicable, describe	8-9	Patients without follow up were not included in the analysis. the control group consisted of healthy individuals (ASA I) who underwent dental implant

		analytical methods taking account of sampling strategy		placement during the same period, matched to the DOAC group primarily by **age** to minimize potential confounding
Participants		(a) Report numbers of individuals at each stage of study—eg, numbers potentially eligible, examined for eligibility, confirmed eligible, included in the study, completing follow-up, and analyzed	10<br>Fig 1a	Fig 1 a
		(b) Give reasons for non-participation at each stage	10	Potentially Eligible 100 patients examined for eligibility-95- 5 patients dropped due to non show for examination Confirmed eligibility- 91 patients included in the study-91- undergone treatment without follow up patient did not continue with treatment completing follow-up, and analyzed - 80- patient did not undergo follow up<br>fig 1a
Descriptive data			10-11 fig 2	Fig 2 table 1-3

Main results		Give unadjusted estimates and, if applicable, confounder-adjusted estimates and their precision (eg, 95% confidence interval). Make clear which confounders were adjusted for and why they were included	10-13 tables 1-3	Postoperative bleeding occurred in 7 of 41 patients in the DOAC group (17.1%) and in 4 of 39 controls (10.3%). The difference was not statistically significant (Fisher's exact test, p = 0.52; OR = 1.80, 95% CI 0.48–6.72)
Other analyses		Report other analyses done—eg analyses of subgroups and interactions, and sensitivity analyses	1-13	significant connection between bleeding and dental implants insertion concomitant with bone grafting. The risk for postoperative bleeding is higher in those patients but it was not significant (X^2=8.652 df=2 p=0.013) The odds ratio for bleeding in the experimental group is 3.47 Risk factors for bleeding in both groups are: age over 75 years old (the odds ratio for bleeding is 3.92) Medically compromised patient (the odds ratio for bleeding is 2.57 in ASA 3 patients

Key results		Summarize key results with reference to study objectives	11-12	results
Limitations		Discuss limitations of the study, taking into account sources of potential bias or imprecision. Discuss both direction and magnitude of any potential bias	14	discussion
Interpretation		Give a cautious overall interpretation of results considering objectives, limitations, multiplicity of analyses, results from similar studies, and other relevant evidence	15	Discussion and conclusions
Generalisability		Discuss the generalisability (external validity) of the study results	15	discussion
Funding		Give the source of funding and the role of the funders for the present study and, if applicable, for the original study on which the present article is based	1	Self funding
